# Increased traffic exposure and negative birth outcomes: a prospective cohort in Australia

**DOI:** 10.1186/1476-069X-10-26

**Published:** 2011-04-01

**Authors:** Adrian G Barnett, Kathryn Plonka, W Kim Seow, Lee-Ann Wilson, Craig Hansen

**Affiliations:** 1 School of Public Health & Institute of Health and Biomedical Innovation, Queensland University of Technology, Kelvin Grove, Queensland, Australia; 2 The University of Queensland, Brisbane, Queensland, Australia; 3 Queensland Health Metro South Health Service District, Brisbane, Queensland, Australia

## Abstract

**Background:**

Pregnant women exposed to traffic pollution have an increased risk of negative birth outcomes. We aimed to investigate the size of this risk using a prospective cohort of 970 mothers and newborns in Logan, Queensland.

**Methods:**

We examined two measures of traffic: distance to nearest road and number of roads around the home. To examine the effect of distance we used the number of roads around the home in radii from 50 to 500 metres. We examined three road types: freeways, highways and main roads.

**Results:**

There were no associations with distance to road. A greater number of freeways and main roads around the home were associated with a shorter gestation time. There were no negative impacts on birth weight, birth length or head circumference after adjusting for gestation. The negative effects on gestation were largely due to main roads within 400 metres of the home. For every 10 extra main roads within 400 metres of the home, gestation time was reduced by 1.1% (95% CI: -1.7, -0.5; p-value = 0.001).

**Conclusions:**

Our results add weight to the association between exposure to traffic and reduced gestation time. This effect may be due to the chemical toxins in traffic pollutants, or because of disturbed sleep due to traffic noise.

## Background

Exposure to air pollution during pregnancy has been shown to increase the risk of negative birth outcomes such as pre-term birth and low birth weight [1-4]. Although the increased risks are relatively small [5-7], the public health implications are large because exposure to some level of air pollution is ubiquitous in urban areas, and pre-term and low weight babies: stay in hospital longer after birth, have an increased risk of death, and are more likely to develop disabilities [8-10].

Many of the estimated associations between air pollution and birth outcomes have relied on the temporal variation in pollution, but pollution also varies spatially [11]. Pollution levels in a city are generally higher in areas with lots of traffic and industrial areas. Temporal studies also rely on a fixed network of pollution monitors, and these monitors can often be far from subjects' homes. Ignoring the spatial variability in pollution therefore introduces a measurement error that may lead to regression dilution [12]. A study in Brisbane showed a clear strengthening of the association between increased pollution and small fetus size when reducing this measurement error by using pollution monitors closer to women's homes [6]. Studies in Spain have attempted to reduce measurement error by restricting analysis to those women who spent more time at home (where their pollution exposure was estimated), and found stronger associations between pollution exposure and fetal growth and birth weight [7,13].

Another reason for taking a spatial approach in this study was the public interest created by our previous study showing restricted fetal development due to increased air pollution exposure in Brisbane, Australia [6]. A common concern was the distance between the home and a busy road at which health effects occurred. This distance also has implications for council authorities looking to build or expand roads. By examining traffic exposure around the home we aimed to find the distance at which the majority of the negative impacts on birth outcomes occurred.

Although levels of air pollution in South East Queensland are low compared with industrial cities, the population's exposure is relatively high due to an outdoor lifestyle and buildings that are highly permeable [14]. Many homes in Queensland are built to capture breezes in order to give relief from high summer temperatures. But this design also increases their exposure to traffic pollution. People living near major roads, and particularly major road junctions where the traffic often stops, will experience the highest levels of exposure.

## Methods

### Cohort of mothers and newborns

We used data from a prospectively recruited cohort of mothers in Logan, Queensland [15]. The cohort was primarily recruited to study the effects of diet and education on child dental health in a relatively low socio-economic area. To achieve this aim the study collected high quality information on birth outcomes and socio-economic status, making it possible for us to examine the effects of traffic pollution on birth outcomes.

Mothers were invited to participate in the study if they: i) attended an antenatal class at the Logan Hospital Maternity Unit, ii) registered to give birth in the maternity ward at Logan Hospital, or iii) attended early parenting classes in the Logan-Beaudesert Health Service District. Mothers were recruited between January 2007 and July 2008. The percentage of mothers approached who agreed to participate was 84%.

The birth outcomes of gestation length (determined using a clinical assessment at birth), birth weight, head circumference and birth length were collected directly from mothers soon after the birth. At a follow-up visit the mothers provided information on their smoking during pregnancy, age, education and parity. We excluded twin births from this analysis as it complicates the assessment of the anthropometric measures. A total of 1,008 women were recruited, however there were four neonatal deaths and six women miscarried.

All women gave informed consent and the study was approved by the Princess Alexandria Human Research Ethics Committee.

### Traffic measures

We used two measures of traffic: the distance to the nearest road and the number of road segments around the home. A road segment is a section of road occurring between two reference points (e.g., between two intersections). We used three road types, which in order of size are: freeways, highways and main roads. A freeway is divided highway without traffic lights or stop signs. A highway is a major road connecting two or more destinations. A main road is smaller than a highway but bigger than a residential street, and may have traffic lights, roundabouts and other junctions. The road data was for 2007, which is within the time period that the mothers were recruited.

**Figure 1:**
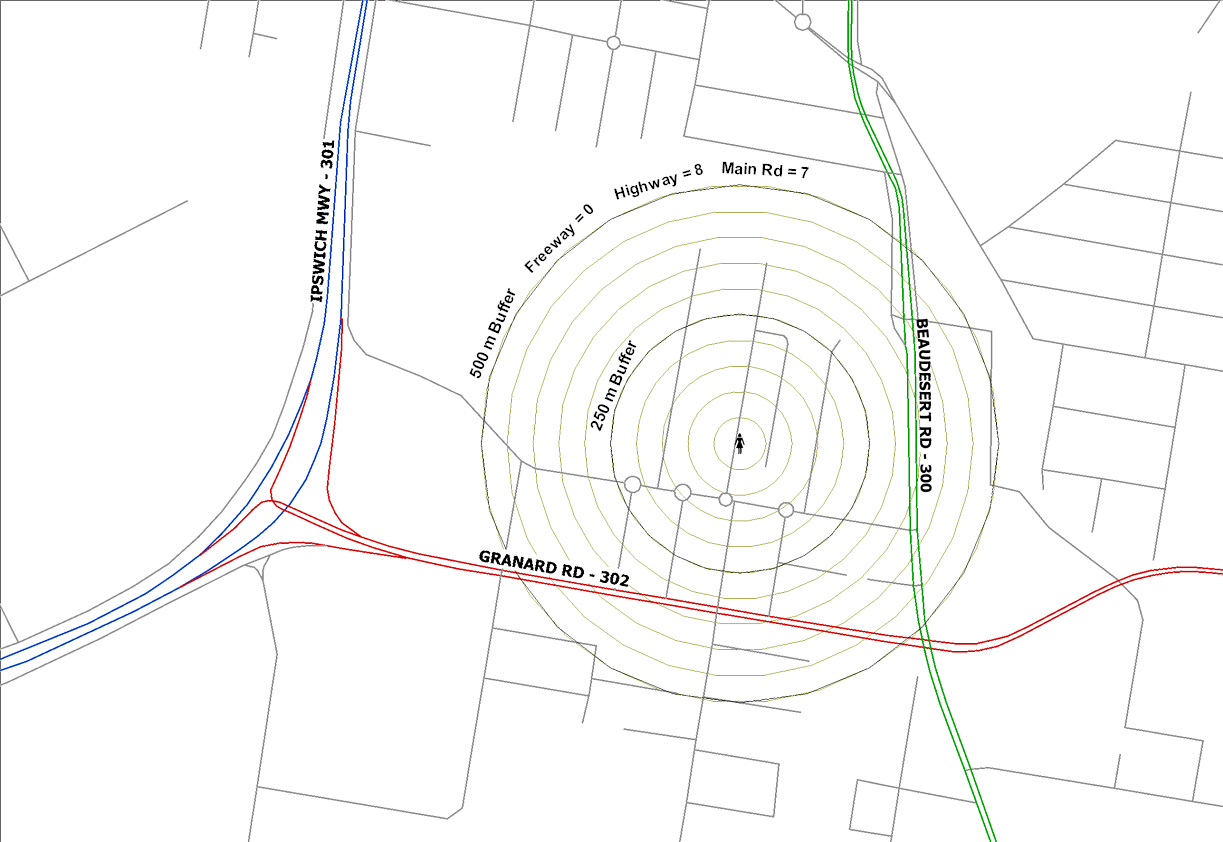
**Example of how the road network around a woman's home was calculated**. Address shown for a random address that was not part of the study. The concentric circles around the home range from 50 to 500 metres. The counts of road segments shown are cumulative.

The participants' addresses were geocoded using MapInfo 9.5 and the Euclidean distance was calculated between participant's residence and the nearest road. Concentric circles with radii 50 to 500 metres in steps of 50 metres were created around each address. The number of road segments in each circle were counted. Figure 1 shows an example of the concentric circles. The largest radius of 500 metres was chosen based on studies that modelled the dispersion of traffic pollution [16,17].

The addresses for 28 mothers (2.7% of all addresses) could not be geocoded because the address information was inadequate (e.g., because a post box was given instead of an actual address). These mothers were excluded from the analyses, leaving 970 mothers in total.

Thirty-one women (3.2% of included mothers) moved house at some time during their pregnancy. For these women the traffic measures were averaged for their two addresses.

### Statistical methods

We used multiple regression models with four dependent variables: gestation (weeks), birth weight (grams), head circumference (centimetres) and birth length (centimetres). We adjusted for gestation when examining birth weight, head circumference and birth length. We analysed all variables on a continuous scale, rather than using categories such as low birth weight (under 2,500 grams) or pre-term birth (before 37 weeks), because of the loss of power caused by categorisation [18].

The independent variables were mother's age, mother's smoking during pregnancy (yes/no), household smoking during pregnancy (yes/no), mother's parity (nulliparous/multiparous), mother's education (primary/high school/TAFE/Tertiary), newborn gender, season of birth and the traffic measures (described below). We controlled for season of birth using a cosinor [19]. As a sensitivity analysis we instead controlled for season of birth using a spline with four degrees of freedom per year, but found no change for the traffic measures and so present the results based on the cosinor.

For all models we assumed that the residuals followed a Normal distribution. We log-transformed gestation to improve the validity of the Normal assumption, and so present the results for gestation on the scale of percent change. We visually checked the residuals for any patterns or large outliers using scatter plots. We also looked for influential observations using the dfbeta statistic [20].

All analyses were made using the freely available R statistical software package version 2.11.1 [21].

### Distance to nearest road

We examined the effect of the distance from the mothers' homes to the nearest road on the four birth outcomes, assuming that distance to road is a proxy for traffic pollution. The concentration of traffic pollutants are spatially non-linear, with the highest levels adjacent to roads and a non-linear decay with increasing distance [17,22]. To model this non-linear exposure we used a spline for the distance to road measures [23]. We used three degrees of freedom for this spline as this gives enough flexibility to model an exponential-like decay in risk. These models were fitted using the "mgcv" R library. The distributions of the distances from roads were strongly positively skewed. We therefore log-transformed the distances so that the regression spline knots were more equally spaced. We checked the sensitivity of all our results to the small number of women who lived far from any roads by repeating the analyses without these women. As there was little difference in the results we show the results for all women. We plot the splines and report their approximate p-values based on the F-distribution that tests the significance of the spline [23, Section 4.8.5].

### Number of road segments

We examined the effect of the number of road segments around the mothers' homes as a proxy of traffic pollution that incorporates traffic volume. We counted the number of roads around the mothers' homes in concentric circles of radii 50 to 500 metres. To find the radius which had the strongest association with birth outcomes we fitted separate models for each radii (10 models per road type). As a comparison we fitted a model without any roads. We then selected the best radius using the Akaike information criterion (AIC) [24]. A difference in the AIC between two models of 0-2 is considered small, whereas differences above 10 are considered large [25].

For descriptive purposes we calculated the Spearman's rank correlation between the number of roads segments at all radii and for all three road types.

### Missing data

Around 10% of mothers had no information on parity, smoking status or education. So as not to lose these women, we randomly imputed any missing responses using the marginal probabilities from the complete sample. For example, 24% of mothers smoked during the pregnancy, so we imputed a positive response to smoking status using a Bernoulli distribution with a "success" probability of 0.24. We repeated the imputation process 10 times (multiple imputation) and combined the results using the R package "mitools" [26].

### Confounding of traffic exposure by socio-economic status

People who live near a major road or in areas with many roads may have a lower socio-economic status than those who live further away in cleaner and more desirable locations. If this is true then our traffic exposure proxies are likely to be confounded by socio-economic status when estimating their effects on birth outcomes. To investigate this we fitted logistic regression models to examine if there was an association between the dependent variables of smoking (of the mother and the household) and tertiary education, and the independent variables of distance to road and the number of road segments. The best radius for the number of road segments was selected using the AIC as described above.

## Results

The basic characteristics of the mothers and newborns are in Table 1. There was a relatively high rate of smoking during pregnancy (24%). Women generally lived closer to a main road than the other two road types, and had more main roads around their home.

**Table 1:** Characteristics of the cohort of 970 women and newborns, Logan, Queensland (June 2006 to December 2008)

Discrete variables	Number missing	N (%)	
	
Mother's smoking during pregnancy = Yes	113	208 (24)	
Household smoking during pregnancy = Yes	110	312 (36)	
Mother's education = Primary	109	10 (1)	
= High school	-	414 (38)	
= TAFE	-	235 (27)	
= Tertiary	-	202 (24)	
Newborn gender = Male	0	460 (47)	
Continuous variables	Number missing	Mean (SD)	Range

Mother's age (years)	11	28.2 (5.7)	15-47
Gestation (weeks)	0	39.6 (1.6)	31.4-42.1
Birth weight (grams)	0	3,453 (549)	1,154-6,000
Head circumference (cm)	32	34.7 (1.6)	29.0-40.0
Birth length (cm)	10	51.4 (2.9)	41.0-63.0

Traffic measures	Number missing	Median (IQR)	Range

Distance to freeway (km)	0	1.9 (2.4)	0.06-33.3
Distance to highway (km)	0	6.5 (6.1)	0.01-20.2
Distance to main road (km)	0	0.6 (0.9)	0.01-7.9
Number of freeway segments within 500 metres	0	0 (0)	0-14
Number of highway segments within 500 metres	0	0 (0)	0-14
Number of main road segments within 500 metres	0	0 (5)	0-31

TAFE = Technical and Further Education, SD = Standard deviation, IQR = Inter-quartile range

**Figure 2:**
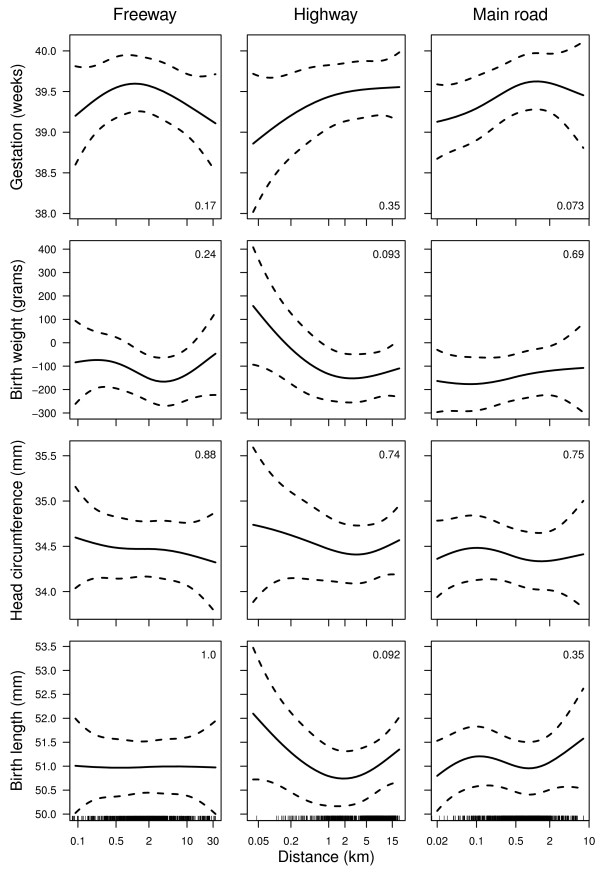
**Associations between birth outcomes and distance to road**. The solid line shows the mean association and the dashed lines the 95% confidence interval. Road distances are on a log scale. The number in the bottom-right or top-right corner is the p-value for the association between distance from road and the birth outcome. The bottom row of plots includes a ''rug'' plot showing the distribution of distances.

Figure 2 shows the estimated birth outcomes depending on distance to the three road types. None of the associations were statistically significant. There was some evidence of shorter gestation times for women living nearer to a major road (p-value = 0.073). The only other marginal associations were a larger birth weight and longer birth length for women living nearer to a highway.

**Figure 3:**
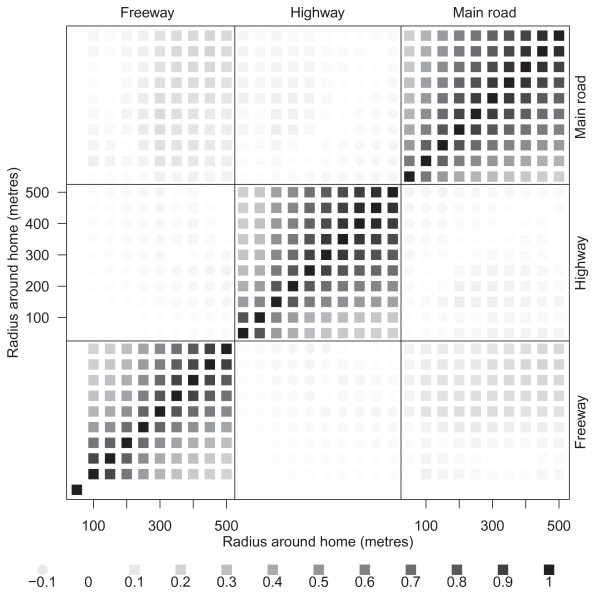
**Spearman's correlations for the number of road segments around the mother's home for radii from 50 to 500 metres**. Stronger correlations have a darker colour. Negative correlations are shown using a circle and positive correlations a square. There were no mothers with a freeway within 50 metres of their home.

Figure 3 shows the correlations between road segments by distance and for the three road types. There were strong correlations for roads of the same type, and weaker correlations between different road types. This lack of correlation between road types encouraged us to examine their effects separately.

**Figure 4:**
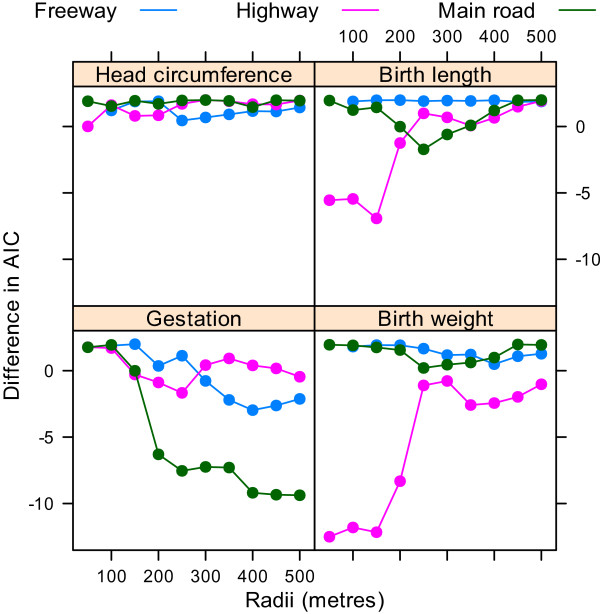
**Differences in the Akaike information criterion for the association between the four birth outcomes and the cumulative number of roads for radii from 50 to 500 metres compared with a model with no road measure**. There were no mothers with a freeway within 50 metres of their home.

Figure 4 shows the difference in the AIC for models using increasing radii compared with a model with no road segments. For the number of highways the best model was for highways within 150 metres of the home for birth length, 250 metres for gestation, and just 50 metres for birth weight. For the number of main roads there was a large improvement in fit at 200 metres, with the best model at 400 metres.

**Figure 5:**
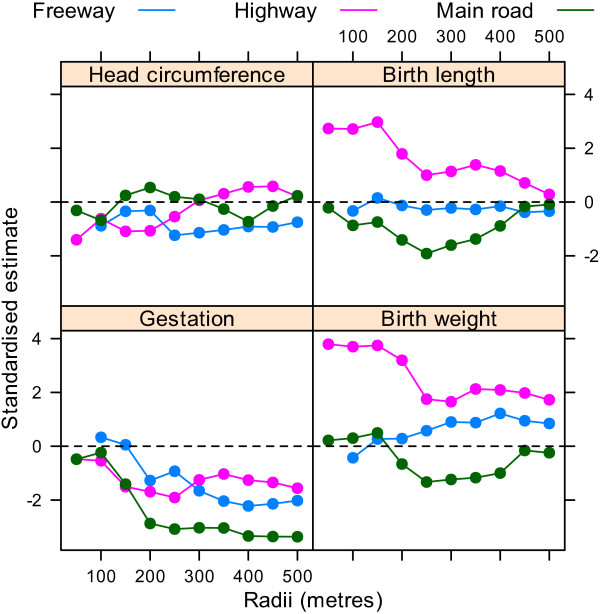
**Standardised estimates for the association between the four birth outcomes and the cumulative number of roads for radii from 50 to 500 metres**. There were no mothers with a freeway within 50 metres of their home.

Figure 5 shows the standardised estimates for the four outcomes and three different road types across the different radii. For birth length and birth weight the effect estimates associated with highways generally moved closer to zero as the radius increased. For gestation the effect estimates for main roads moved further from zero as the radius increased.

**Figure 6:**
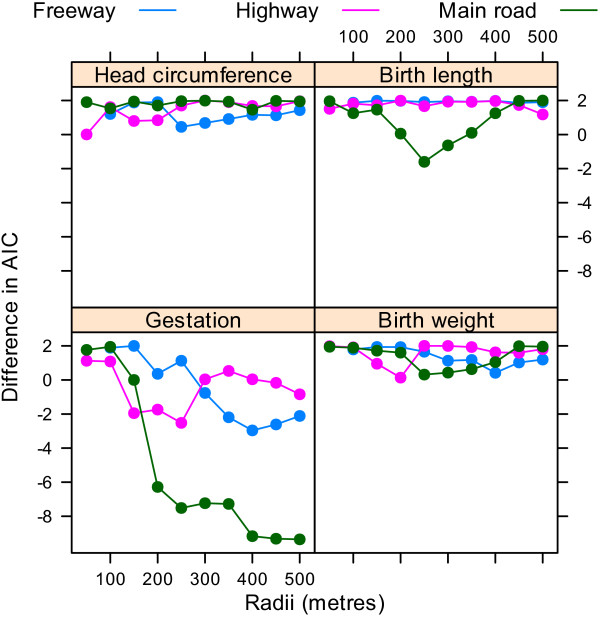
**Differences in the Akaike information criterion for the association between the four birth outcomes and the cumulative number of roads for radii from 50 to 500 metres compared with a model with no road measure**. Results after removing one influential mother. There were no mothers with a freeway within 50 metres of their home.

**Figure 7:**
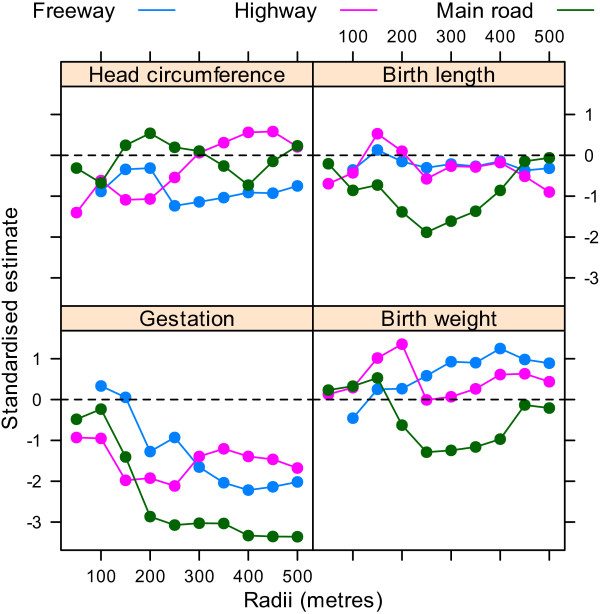
**Standardised estimates for the association between the four birth outcomes and the cumulative number of roads for radii from 50 to 500 metres**. Results after removing one influential mother. There were no mothers with a freeway within 50 metres of their home.

Using the dfbeta statistic there was a large influential observation for the analyses of birth length and birth weight analyses dependent on highways. This was the mother with the largest birth weight (6,000 g) and longest birth length (63 cm), and the equal largest number of highways within 50 metres (1 highway). After removing this one mother the changes in the AIC looked quite different as there was no longer a big improvement in model fit at the shortest radii (Figure 6). The standardised estimates were also quite different, as the large increases in birth weight and length associated with highways were no longer present (Figure 7). There were no large influential observations for any of the other associations.

Table 2 shows the estimated changes in birth outcomes based on a 10 unit increase in road numbers. These results exclude the one influential mother identified above. We used a radius of 250 metres for birth length and 400 metres for the other three outcomes based on the AIC in Figure 6. The most striking results were a reduction in gestation time associated with increased freeways (4.4% shorter) and main roads (1.1% shorter).

**Table 2:** Associations between the number of road segments around the mothers' homes and the four birth outcomes

Birth outcome (scale)	Road type	Radius (metres)	Estimate	95% CI	P-value
Gestation (%)	Freeway	400	-4.4	-8.1,-0.5	0.03
	Highway	400	-2.5	-5.9,1.0	0.16
	Main road	400	-1.1	-1.7, -0.5	0.001
Birth weight (g)	Freeway	400	288.2	-164.2, 740.6	0.21
	Highway	400	126.5	-278.9, 531.9	0.54
	Main road	400	-37.4	-113.1, 38.2	0.33
Birth length (cm)	Freeway	250	-0.10	-0.77, 0.57	0.76
	Highway	250	-0.13	-0.59, 0.32	0.57
	Main road	250	-0.08	-0.16, 0.003	0.06
Head circumference	Freeway	400	-0.70	-2.20, 0.80	0.36
(cm)	Highway	400	0.39	-0.90, 1.62	0.57
	Main road	400	-0.09	-0.33, 0.15	0.47

Estimates shown for a 10 unit increase in roads. Results after excluding one influential mother.

CI = confidence interval

We found some potential confounding of the association between traffic and birth outcomes by socio-economic status. There was an association between distance to freeway and odds of smoking at both a mother and household level (Table 3). Mothers who lived further from a freeway were less likely to smoke and less likely to be exposed to household smoke.

**Table 3:** Associations between the two road measures and three indicators of social class (mother and household smoking, and mother's education)

Traffic measure	Dependent variable	Road type	OR	95% CI	P-value
Distance to road (km)	Mother's	Freeway	0.95	0.91,1.00	0.04
	smoking	Highway	0.97	0.94, 1.01	0.12
		Main road	1.01	0.83, 1.23	0.94

	Household	Freeway	0.94	0.91, 0.98	0.004
	smoking	Highway	0.99	0.96, 1.03	0.70
		Main road	1.14	0.97, 1.35	0.12

	Mother's	Freeway	0.99	0.95, 1.03	0.57
	education	Highway	1.01	0.98, 1.05	0.47
		Main road	1.10	0.92, 1.32	0.29

Number of road segments within 400 metres	Mother's smoking	Freeway	0.83	0.59, 1.17	0.29
		Highway	1.00	0.82, 1.24	0.96
		Main road	1.02	0.98, 1.06	0.31

	Household	Freeway	0.91	0.72, 1.15	0.44
	smoking	Highway	1.10	0.92, 1.31	0.29
		Main road	0.99	0.96, 1.03	0.75

	Mother's	Freeway	1.03	0.83, 1.29	0.77
	education	Highway	1.08	0.89, 1.31	0.43
		Main road	1.01	0.97, 1.05	0.53

Odds ratios are on a scale of per km for distance, and per segment for the number of roads.

OR = odds ratio, CI = confidence interval

## Discussion

Our results add weight to the association between exposure to traffic pollution and reduced gestation time [2,4]. A recent systematic review in this area found that six out of seven studies found an association between exposure to traffic and pre-term birth [3]. However, the biological route remains unknown. A study in the Czech Republic found an association between exposure to particulate matter (PM) and increased T cells (CD3^+ ^and CD4^+^) and decreased B cells and natural killer cells in placental blood [27]. Exposure to particulate matter has also be shown to increase oxidative stress [28] which has been linked to pre-term birth [29].

As well as the increased exposure to chemical pollutants caused by traffic, traffic also creates noise which may increase stress and disturb sleep. Disturbed sleep during pregnancy may be a risk factor for adverse birth outcomes [30]. Traffic noise is often greatest at junctions where vehicles brake and accelerate. This stopping and starting also means that junctions have some of the highest levels of air pollution, particularly those junctions where traffic jams occur. This makes it difficult to separate the effects of air and noise pollution.

The plot of the AIC indicated that the negative effects of traffic on gestation were largely associated with main roads within 400 metres of the home (Figure 6), with much of the effect for roads within 200 metres. The distance that air pollutants can travel is partly dependent on wind direction and barriers. A study in the Netherlands found that concentrations of air pollutants in and outside schools near motorways were significantly associated with distance, traffic density and composition, and percentage of time downwind [31]. A study in California found that ultrafine particle concentrations measured at 300 metres downwind from a freeway were indistinguishable from background concentrations [22]. A study in Brisbane found that particulate matter from traffic travelled up to 375 metres, although concentrations were much higher closer to the road [16]. A study in Victoria found that nitrogen dioxide and PM_10 _concentrations were higher for homes near busy roads (within 150 metres) compared with roads that were more than 300 metres away [32]. Our results that show that the majority of the negative effect within 200 metres, but with effects up to 400 metres, are therefore consistent with these monitoring studies.

The distance that traffic noise can travel is dependent on the frequency of the noise, for example, the low frequency rumbling of heavy goods vehicles compared with the high frequency squeaking of brakes. Noise pollution can be blocked by sound barriers. We were unable to find any studies that gave the maximum distance for the effects of traffic noise. Interestingly the Queensland department of Main Roads is currently installing free air conditioning or mechanical ventilation to homes with 300 metres of the Pacific motorway just to the south of the study area [33].

We failed to find any association with negative birth outcomes and distance to road (Figure 2), but did find associations using the number of roads (Table 2). This may be because the number of roads are a better proxy of traffic volume than distance to road, and traffic volume is the key determinant of both air and noise pollution.

We failed to find any association between traffic and the three anthropometric birth outcomes (weight, length and head circumference), but these associations were adjusted for gestation. So pollution is still likely to negatively influence birth size, but via the indirect route of a shortened gestation.

The effects of the number of roads on gestation were small, with a mean 4.4% decrease in gestation due to 10 more freeways around the home, and a mean 1.1% decrease for ten more main roads. For a gestation of 40 weeks this would be a reduction to 38.2 weeks for freeways and 39.6 weeks for main roads. However, these small reductions in gestation may have big implications for later life, as healthier babies have healthier childhoods and adulthoods [8-10].

### Limitations

This study was relatively small compared with others in the field, but unlike some of the large retrospective cohort studies we had detailed information on potential confounders such as smoking. Because of the small sample size we did not examine the effects of pollution on spontaneous abortion or stillbirth, but feel that this is an important area for future research.

Our measures of traffic exposure were relatively crude, and did not include a measure of the number of vehicles or the types of vehicles. Our method assumes that the effect of each road type is constant across the study area. Other more sophisticated estimates of traffic, that would reduce exposure misclassification, are a weighted road density [34] or land use regression [35,36]. We also did not consider the measurement error in exposure due to the individual characteristics of the mothers. For example, accounting for those who spent more time at home, or those who had air conditioning. Despite these measurement errors we were still able to show a strong association between traffic and adverse birth outcomes. We think it is likely that more accurate estimates of exposure would lead to stronger associations between traffic exposure and birth outcomes because of the regression dilution bias [12].

We found some evidence of confounding by socio-economic status (Table 3), with mother's who lived closer to a freeway being more likely to smoke and more likely to experience household smoke. Although we controlled for education and smoking in all our models, there is still a danger that the detrimental effect of traffic exposure is due to residual confounding with socio-economic status. Other studies in this area have been mixed with a study in France showing an association between traffic exposure and lower socio-economic status [37], whilst a study in Italy found the reverse, with higher traffic associated with higher a socio-economic status [38].

We found one very influential mother who we removed according to the large dfbeta value and noticeable effect on the results (compare Figures 5 and 7). The influence of this one mother was mostly concerned with birth weight and birth length (for which her baby had unusually high values). We gave the results with and without this mother, but have more faith in the results excluding this mother as we feel that the results of a study of nearly 1,000 mothers should not be dominated by the results from one mother.

## Conclusions

Pregnant women should reduce their exposure to traffic. A reduction in traffic emissions, whether through improved vehicle technology or increased public transport use, would have immediate health benefits by giving children a better start to life.

## List of abbreviations

AIC: Akaike information criterion; CI: confidence interval; OR: odds ratio; PM: particulate matter.

## Competing interests

The authors declare that they have no competing interests.

## Authors' contributions

AB led the study, ran the statistical analyses and wrote the first draft. KP and WKS designed and oversaw the data collection. LAW created the GIS data. CH provided expertise on the health effects of pollution. All authors contributed to drafts of the paper and approved the final manuscript.
